# Transcranial magnetic stimulation as a new approach in medication overuse headache: a pilot study

**DOI:** 10.1186/1129-2377-14-S1-P168

**Published:** 2013-02-21

**Authors:** A Granato, S Musho Ilbeh, F Trovò, M Borelli, G Granello, M Semenic, F Monti, G Pizzolato

**Affiliations:** 1 Department of Medical, Surgical and Health Sciences, Headache Centre, University of Trieste, Trieste, Italy; 2 Department of Mathematics and Geosciences, University of Trieste, Trieste, Italy

## Background

Repetitive TMS (rTMS) is effective in migraine prophylaxis.

## Introduction

To study the efficacy of high-frequency rTMS in medication overuse headache (MOH).

## Methods

A prospective, double-blind, randomized, placebo-controlled trial on patients suffering from MOH consecutively presenting in a six-month period in the Headache Centre of Trieste was performed. Patients were randomized into the rTMS or the sham-TMS group. Treatment consisted of 10 consecutively TMS sessions delivered on left dorsolateral prefrontal cortex, each session being 10 trains of 2-s duration, separated by 30-s pause, 20 Hz frequency, 100% motor threshold intensity. Demographic and clinical information, MIDAS score, headache days (HD), hours of headache (HH), and symptomatic drugs (SD) in the 3 months before (t1), and in the first (t2) and second month (t3) after stimulation were analysed using SPSS 14.0.

## Results

We enrolled 8 patients (7 F, 1 M; mean age 44 ± 11), four patients undewent rTMS and four sham-TMS. All patients were migraineurs without aura as initial primary headache. We found, in both rTMS and sham-TMS group, no significant difference between the 3 months before and the 2 months after stimulation (rTMS: HD= 22 ± 6 t1 vs 22 ± 11 t2 vs 19 ± 14 t3, HH= 223 ± 205 t1 vs 219 ± 198 t2 vs 205 ± 196 t3, SD= 22 ± 10 t1 vs 18 ± 7 t2 vs 16 ± 8 t3; sham-TMS: HD= 22 ± 5 t1 vs 12 ± 6 t2 vs 13 ± 8 t3, HH= 180 ± 117 t1 vs 99 ± 73 t2 vs 97 ± 28 t3, SD= 22 ± 10 t1 vs 16 ± 3 t2 vs 17 ± 4 t3). MIDAS score significantly reduced in rTMS group at a three-month evaluation (111 ± 29 vs 42 ± 27; p=0.03).

## Conclusions

Our preliminary data suggest that high-frequency rTMS is not useful to treat MOH, however the small sample does not allow to draw safe conclusions.
